# USE OF PSYCHIATRIC RISK FACTORS IN PREDICTING 10 YEAR CARDIOVASCULAR RISK FOR CHINESE PATIENTS WITH SCHIZOPHRENIA

**DOI:** 10.1093/geroni/igad104.2450

**Published:** 2023-12-21

**Authors:** Jennifer Yee-man Tang, Hao Luo, Gary K K Lau, Edwin H M Lee, Pengcheng Wang, Ian C K Wong, Terry Lum, Gloria Hoi-Yan Wong

**Affiliations:** The Chinese University of Hong Kong, Hong Kong, Hong Kong; The University of Hong Kong, Hong Kong, Hong Kong; The University of Hong Kong, Hong Kong, Hong Kong; The University of Hong Kong, Hong Kong, Hong Kong; The University of Hong Kong, Hong Kong, Hong Kong; The University of Hong Kong, Hong Kong, Hong Kong; The University of Hong Kong, Hong Kong, Hong Kong; The University of Hong Kong, Hong Kong, Hong Kong

## Abstract

Patients with schizophrenia are known to have excessive cardiovascular morbidity. Traditional Framingham risk prediction models do not consider additional risk attributable to factors related to psychiatric illness and treatment. The Framingham risk scores also tend to over-estimate cardiovascular risk in Chinese cohorts. This study aimed to develop a 10-year cardiovascular risk prediction model using routine clinical data from an electronic health database in Hong Kong. We identified a cohort of 39,449 patients who were diagnosed with schizophrenia between Year 2005 and 2015 and had no history of cardiovascular disease (CVD). Cox proportional hazards regression models were employed to determine psychiatric and clinical risk factors influencing the timing of incident CVD over 10 years. Psychiatric variables of interest included comorbid psychiatric disorders, duration of schizophrenia, use of antidepressant, use of first- or second-generation antipsychotics (FGA & SGA) at baseline and duration of exposure to FGA and SGA. The main analysis showed that comorbid substance use-related disorders (HR = 1.52; 95% CI 1.22 – 1.90; P < 0.001) increased CVD risk and each year of cumulative exposure to FGA increased CVD risk by 2% (HR = 1.02; 95% CI 1.01 – 1.04; P = 0.001). The use of antidepressant predicted a higher CVD risk in female patients (HR 1.19; 95% CI 1.02 – 1.38; P = 0.028). Our risk model predicted that 20.6% of patients had a CVD risk ≥ 20% over 10 years. Our risk models may offer an accessible clinical tool to help monitor cardiovascular risk in patients with schizophrenia.

